# Effect of the novel anti-NGF monoclonal antibody DS002 on the metabolomics of pain mediators, cartilage and bone

**DOI:** 10.3389/fphar.2024.1396790

**Published:** 2024-08-12

**Authors:** Dandan Jin, Haoyi Yang, Zhiyou Chen, Yuxin Hong, Hehua Ma, Zhenzhen Xu, Bei Cao, Fei Fei, Yuwen Zhang, Weitao Wu, Lei Tang, Runbin Sun, Chunhe Wang, Juan Li

**Affiliations:** ^1^ Department of Phase I Clinical Trials Unit, Nanjing Drum Tower Hospital Affiliated to Nanjing University of Chinese Medicine, Nanjing, China; ^2^ Department of Phase I Clinical Trials Unit, Nanjing Drum Tower Hospital, Affiliated Hospital of Medical School, Nanjing University, Nanjing, China; ^3^ Department of Phase I Clinical Trials Unit, China Pharmaceutical University Nanjing Drum Tower Hospital, Nanjing, China; ^4^ Dartsbio Pharmaceuticals Ltd., Zhongshan, Guangdong, China

**Keywords:** metabolomics, pain, aromatic amino acids, biomarkers, anti-ngf drugs

## Abstract

The anti-nerve growth factor antibody class of drugs interrupts signaling by blocking NGF binding to TrkA receptors for the treatment of pain; however, this target class of drugs has been associated with serious adverse effects in the joints during clinical trials. DS002 is a novel anti-nerve growth factor antibody drug independently developed by Guangdong Dashi Pharmaceuticals. The main purpose of this study is to explore the correlation between DS002 and pain as well as cartilage and bone metabolism with the help of metabolomics technology and the principle of enzyme-linked reaction, and to examine whether DS002 will produce serious adverse effects in joints caused by its same target class of drugs, in order to provide more scientific basis for the safety and efficacy of DS002. Our results showed that DS002 mainly affected the metabolism of aromatic amino acids and other metabolites, of which six metabolites, l -phenylalanine, 5-hydroxytryptophan, 5-hydroxytryptamine hydrochloride, 3-indolepropionic acid, kynuric acid, and kynurenine, were significantly altered, which may be related to the effectiveness of DS002 in treating pain. In addition, there were no significant changes in biological indicators related to cartilage and bone metabolism *in vivo*, suggesting that DS002 would not have a significant effect on cartilage and bone metabolism, so we hypothesize that DS002 may not produce the serious adverse effects in joints caused by its fellow target analogs. Therefore, the Anti-NGF analgesic drug DS002 has the potential to become a promising drug in the field of analgesia, providing pain patients with an efficient treatment option without adverse effects.

## Graphical Abstract

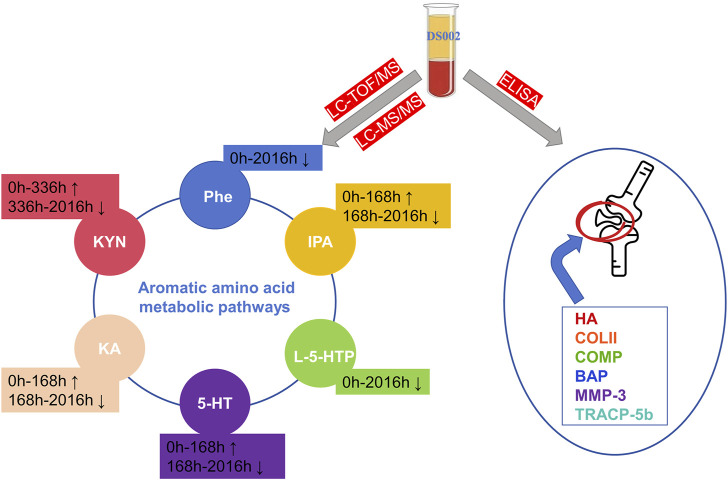

## 1 Introduction

Chronic pain is an unsolved global healthcare problem that remains a major cause of suffering, disability, and substantial economic burden worldwide (Cohen et al., 2021). It is estimated that the rate of chronic pain in humans is as high as 20% each year (Dahlhamer et al., 2018). The International Association for the Study of Pain (IASP) defines pain as unpleasant sensory and emotional experiences related to or similar to actual or potential tissue damage (Raja et al., 20220). Pain can be categorized as acute or chronic based on the duration and nature of its occurrence. Modern medical research has proven that acute pain is a symptom that goes away as the disease gets better; However, chronic pain usually lasts for more than 1 month or even 3 months, and this pain is no longer a symptom, but should be treated as a disease. The occurrence of pain is caused by many factors. In addition to the impact of injury or disease, there are physical perception, psychological and emotional changes, genetic factors, etc. Due to the complexity of pain, the results of the current treatment of chronic pain are unsatisfactory (The, 2021). Currently available analgesics do not meet treatment goals. For example, opioids and non-steroidal anti-inflammatory drugs (nsaids), two commonly used analgesics in clinical practice, have different side effects in chronic pain. The main adverse reactions induced by opioids include analgesic tolerance, addiction and physical tolerance, nausea and vomiting, constipation, and respiratory depression (Paul et al., 2021). The long-term use of NSAIDs can produce gastric bleeding and cardiovascular-related adverse reactions (Trelle et al., 2011). The different side effects greatly limit the widespread use of these two classes of drugs. Therefore, there is an unmet need for effective analgesic drugs that are safer and more tolerable than currently available treatments.

Nerve growth factor (NGF) was first discovered by Rita Levi-Montalcini (Levi-Montalcini, 1952). NGF in the body is both an essential protein and an important pain-producing substance (Hirose et al., 2016). It has been found that NGF binding can be two types of receptors (Landreth and Shooter, 1980), the protomyosin receptor kinase a (TrkA), which has a high affinity for NGF, and the p75 neurotrophic factor receptor (p75NTR), which has a low affinity for NGF. NGF is secreted by inflammatory cells in damaged tissues and by Schwann cells in damaged nerves (Thacker et al., 2007; Basbaum et al., 2009). When tissue injury occurs, NGFs are abundantly expressed at the site of injury and bind to TrkA receptors on the cell membranes of sensory nerve endings, which in turn transmits pain signals, ultimately leading to the onset of pain. Therefore, in cases where an inflammatory response persists with damaging pain, such as low back pain, back pain, autoimmune diseases - rheumatism, etc., or in cases of neuropathic pain, NGF induces sensitization of the peripheral and central systems, which can lead to nociceptive hypersensitivity and anisocoria, and analgesics that block NGF/TrKA signaling can be very effective in these pathologies (Hirose et al., 2016). In recent years, researchers have also identified it as one of the key targets for drugs to treat chronic pain. Currently, the main NGF/TrkA inhibitor drugs are anti-NGF antibodies and TrkA activity inhibitors (Hirose et al., 2016). This study will focus on a new anti-NGF antibody, which have shown promising results in clinical studies and are expected to provide new clinical treatment strategies for chronic pain in the future.

Metabolomics is the quantitative and qualitative analysis of all small molecules in a biological sample at a certain time and under certain conditions. It is a accredited methodology with more than 20 years of practical experience. At present, metabolomics has been extended to many disciplines (Jeyarajah et al., 2006; Wishart, 2016; González-Domínguez et al., 2020). Metabolomics technologies have improved the mechanistic understanding, diagnosis, and treatment of various diseases by utilizing biological samples from humans and animal and cellular models of human diseases. Although the relationship between metabolomics and pain may be complex because the occurrence of pain is accompanied by multiple physiological symptoms, these issues have the potential to manifest through the level of metabolomics (Miettinen et al., 2021), which will facilitate the development of mechanism-based pain diagnostics to support mechanism-based therapeutic approaches. Metabolomics research has the potential to advance these goals because it can discover pain biomarkers to improve pain treatment options, facilitate drug development progress, and ultimately reduce the burden of chronic pain.

DS002 is an anti-NGF monoclonal antibody developed by Tashi Pharmaceutical (Guangdong) Co. Ltd. It interrupts the signal transmission by blocking the binding of NGF to the TrkA receptor, making it unable to stimulate the corresponding sensory neurons, and finally achieving an analgesic effect. Currently, a phase I clinical trial of DS002 has been completed and published in our laboratory (Ma et al., 2022). This study aims to explore the effects of DS002 on pain, cartilage and bone metabolism-related markers, and the changes in metabolic profile *in vivo*, to provide more evaluation basis for the drug development of DS002.

## 2 Methods

### 2.1 Sample collection

The study samples were obtained from the Phase I clinical Trial Center of Nanjing Drum Tower Hospital. Blood samples from 48 healthy subjects were enrolled in this study and divided into seven different DS002 dose groups (0.5, 1, 2, 4, 7, 12, 20 mg), and 432 blood samples were collected. The samples used in this study were PK blood samples at 0 h and D4 (72 h), D8 (168 h), D15 (336 h), D29 (672 h), D43 (1008 h), D57 (1344 h), D85 (2016 h), D113 (2688 h) after administration. The collected blood samples were used to determine the changes in serum metabonomics before and at different time points after administration, and the changes of cartilage and bone metabolism-related indexes before and at different time points after administration were determined by enzyme-linked immunosorbent assay (ELISA). This study (CTR20210155) was approved by the Ethics Committee of Drum Tower Hospital, the Affiliated Hospital of Nanjing University Medical School.

### 2.2 Subjects

The project included healthy male and female subjects aged 18–45 years, with female subjects not pregnant or breastfeeding. Enrolled subjects have a body mass index (BMI) of 19.0–26.0 kg/m^2^ and weigh ≥50 kg for males and ≥45 kg for females. Subjects agree to have no fertility or sperm/egg donation plans and to voluntarily use a medically recognised and effective form of contraception for the duration of the trial up to 6 months after dosing. Subjects are sufficiently aware of the content, objectives and characteristics of the study to be able to complete the study as planned and are willing to serve as subjects and are able to sign an informed consent form.

Subjects were excluded if they met any one of the following exclusion criteria: previous history of allergy or hypersensitivity; subjects with a history of osteoarthritic disease, a history of peripheral or autonomic neuropathy, a history of sensory abnormalities and hypoesthesia, a disease or history of unexplained spontaneous bleeding and/or post-traumatic hemorrhage with more than one bleed, a history of thyroid dysfunction or thyroid hormone abnormality, a history of a malignant neoplasm or a history of malignant neoplasms, an individual with an inherited immunodeficiency, or a family history of inherited immunodeficiencies; smokers, alcoholics or drug users; subjects should not take any prescription drugs, over-the-counter medications, or natural health supplements for 14 days prior to screening.

### 2.3 Sample preparation, instrument conditions and data analysis of metabolomics

The specific steps of sample processing were as follows: take 100 μL serum and add 400 μL methanol solution containing isotope internal standard. The samples were shaken for 3 min at 18,000 rpm*10 min and centrifuged at 4°C. 400 μL of the supernatant was transferred to an EP tube and centrifuged again in the same way. 100 μL of the supernatant was taken and 10 μL of the sample was injected. An equal volume of all samples was mixed to prepare quality control samples (QC). QC samples are part of the system regulation and quality control process. The processing and testing of QC samples are similar to those of the analyzed samples, which helps to obtain reliable and high-quality metabolomics data.

LC-TOF/MS instrument conditions:the chromatographic column was Waters HSS T3 (2.1 mm × 100 mm, 1.8 um), the flow rate was 0.3 mL/min, the column temperature was 40°C, and the mobile phase was A: water phase (0.1% formic acid-water). B: organic phase (acetonitrile), MS scanning range: 50 to 1,200 Da. LC-MS/MS instrument conditions: the chromatographic column was Waters HSS T3 (2.1 mm × 100 mm, 1.8 um), the flow rate was 3 mL/min, the column temperature was 40°C, the mobile phase A was 0.1% formic acid water, and the mobile phase B was 0.1% formic acid acetonitrile. Gradient elution: mobile phase B was 10% (0.00–1.00 min), 10%–90% (1.00–9.00 min), 90% (9.00–12.00 min), 90%–10% (12.00–12.10 min), 10% (12.10–15.00 min), the injection volume was 5 uL per injection.

Based on the metabolomics data obtained by LC-TOF/MS, the raw data were converted into mzXML format by MSconvert software. Subsequent analyses were performed using the tidymass package installed in the R project (version >4.2.2). GraphPad Prism 9.0.0 software was used for statistical analysis based on the metabolomics data obtained by LC-MS/MS. Data were expressed as mean and standard deviation, and differences between groups were analyzed by one-way analysis of variance (ANOVA). Differences between all groups were compared first, and then between two pairs. *p* < 0.05 was considered significant in all statistical tests.

### 2.4 ELISA method and data analysis

The three kits of hyaluronic acid (HA), cartilage oligomeric matrix protein (COMP), and collagen type II (COL2α1) used in this study were purchased from Signalway Antibody (SAB, United States). The three kits of matrix metalloproteinase-3 (MMP-3), human bone-derived alkaline phosphatase (BAP), and tartrate-resistant acid phosphatase (TRACP-5) were purchased from Shanghai Jining Industrial Co., LTD. Sample testing procedures were performed exactly according to the instructions included with each kit. The data were analyzed by GraphPad Prism 9.0.0 software.

## 3 Results

### 3.1 Non-targeted metabolomics to screen small molecule metabolites related to pain

**FIGURE 1:**
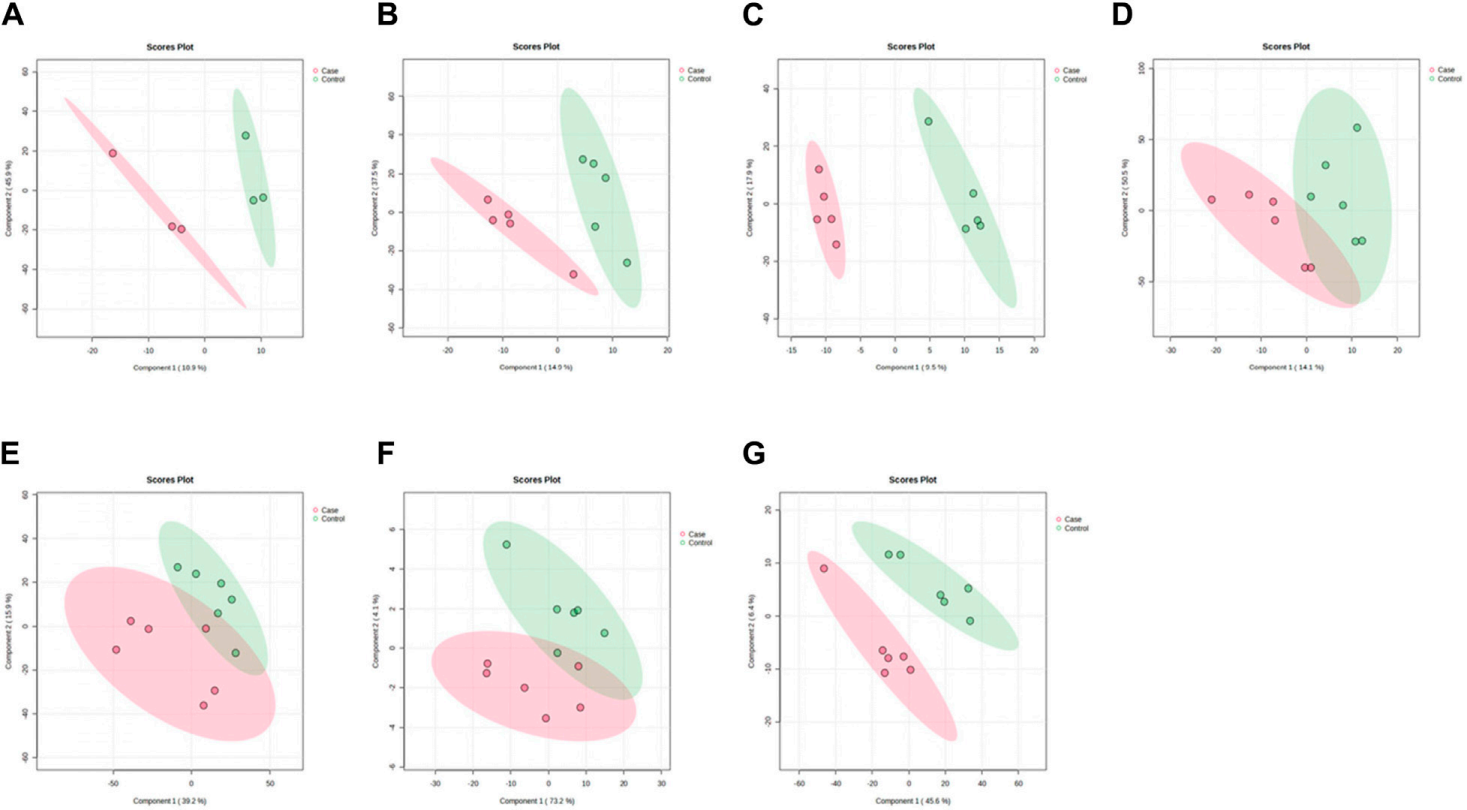
PLSDA plots of samples in ESI + mode before and after administration in different dose groups. The doses were 0.5 mg panel **(A)**, 1 mg panel **(B)**, 2 mg panel **(C)**, 4 mg panel **(D)**, 7 mg panel **(E)**, 12 mg panel **(F)**, and 20 mg panel **(G)**.The red color represents the serum sample collected at 168 h after administration, which is the post-administration. The green represents the serum sample collected at 0 h before administration, which is the pre-administration.

**FIGURE 2:**
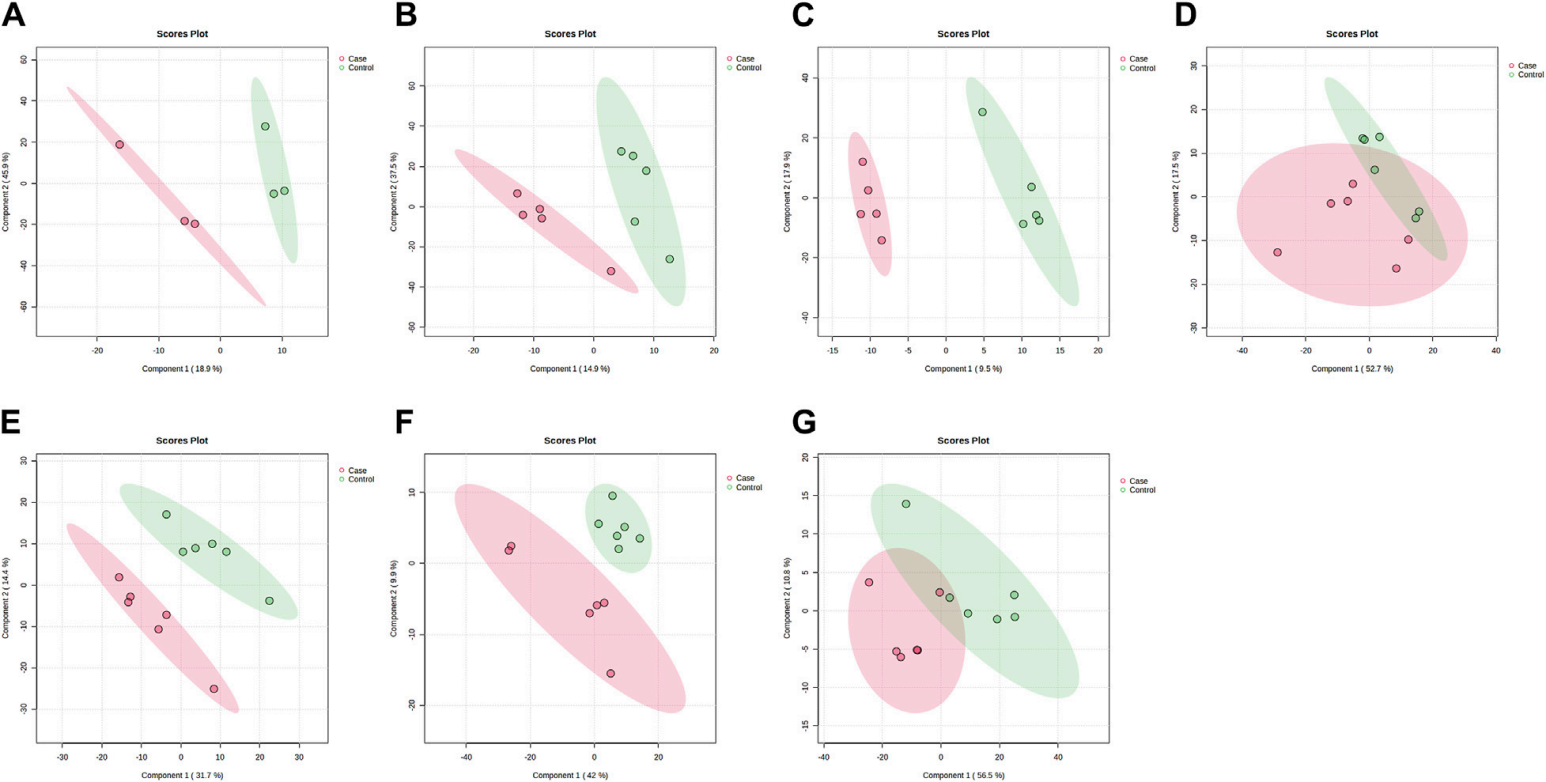
PLSDA plots of samples in ESI-mode before and after administration in different dose groups. The doses were 0.5 mg panel **(A)**, 1 mg panel **(B)**, 2 mg panel **(C)**, 4 mg panel **(D)**, 7 mg panel **(E)**, 12 mg panel **(F)**, and 20 mg panel **(G)**. The red dots represent the serum samples collected at 168 h after administration, which is the post-administration. The green dots represent the serum samples collected at 0 h before administration and are the pre-administration.

**FIGURE 3:**
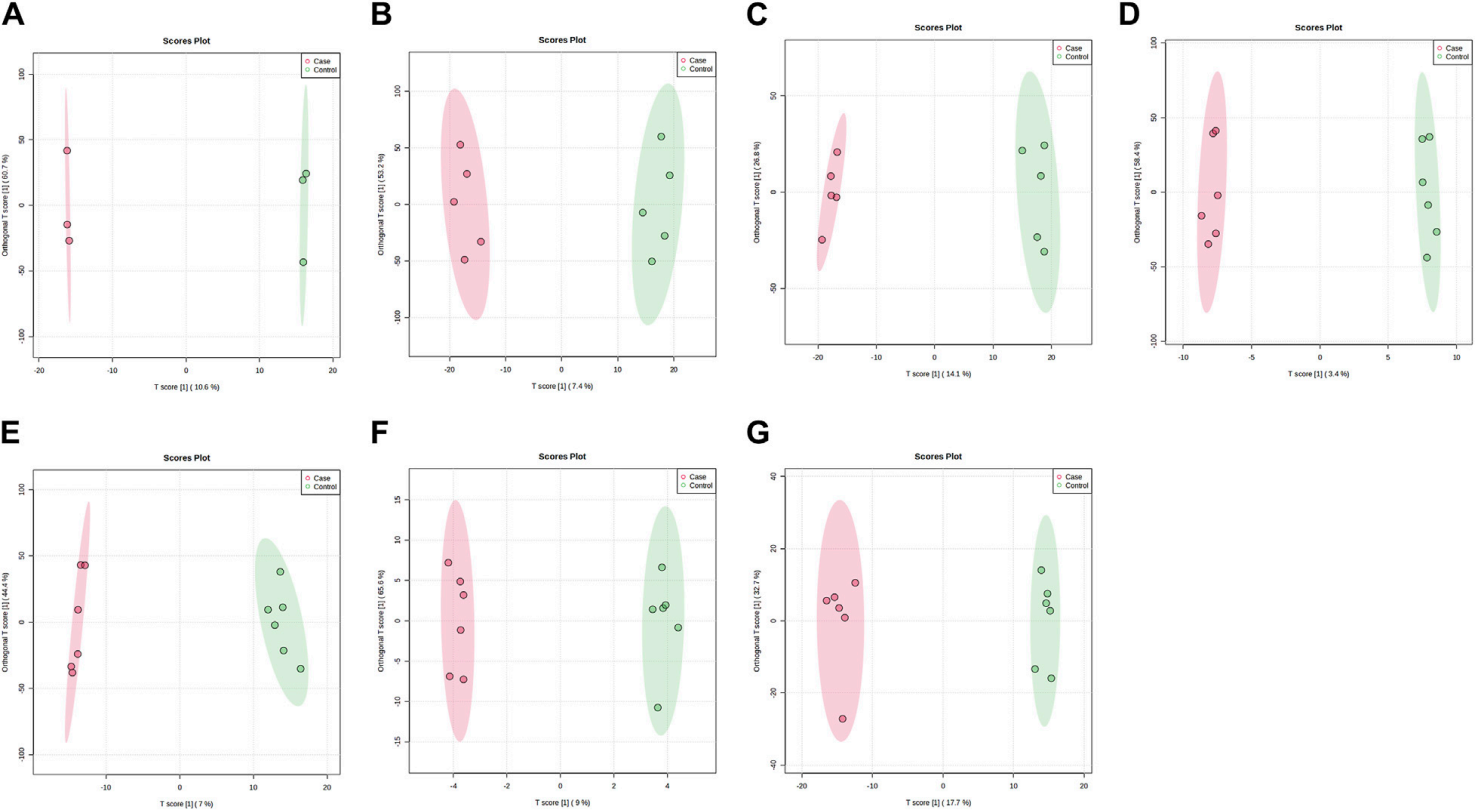
OPLS-DA plots of samples in ESI + mode before and after administration in different dose groups. The doses were 0.5 mg panel **(A)**, 1 mg panel **(B)**, 2 mg panel **(C)**, 4 mg panel **(D)**, 7 mg panel **(E)**, 12 mg panel **(F)**, and 20 mg panel **(G)**. The red dots represent the serum samples collected at 168 h after administration, which is the post-administration. The green dots represent the serum samples collected at 0 h before administration and are the pre-administration.

**FIGURE 4:**
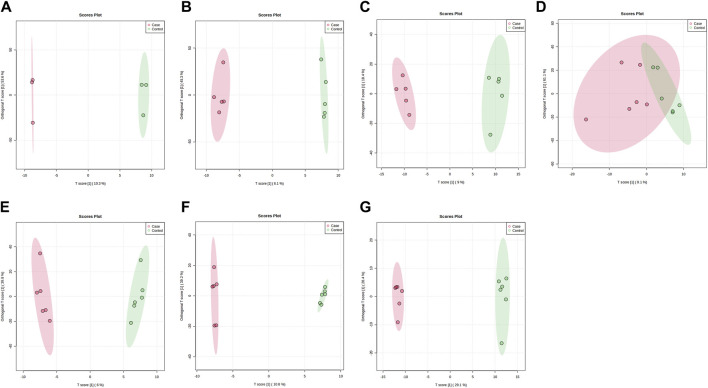
OPLS-DA plots of samples in ESI-mode before and after administration in different dose groups. The doses were 0.5 mg panel **(A)**, 1 mg panel **(B)**, 2 mg panel **(C)**, 4 mg panel **(D)**, 7 mg panel **(E)**, 12 mg panel **(F)**, and 20 mg panel **(G)**. The red dots represent the serum samples collected at 168 h after administration, which is the post-administration. The green dots represent the serum samples collected at 0 h before administration and are the pre-administration.

**FIGURE 5:**
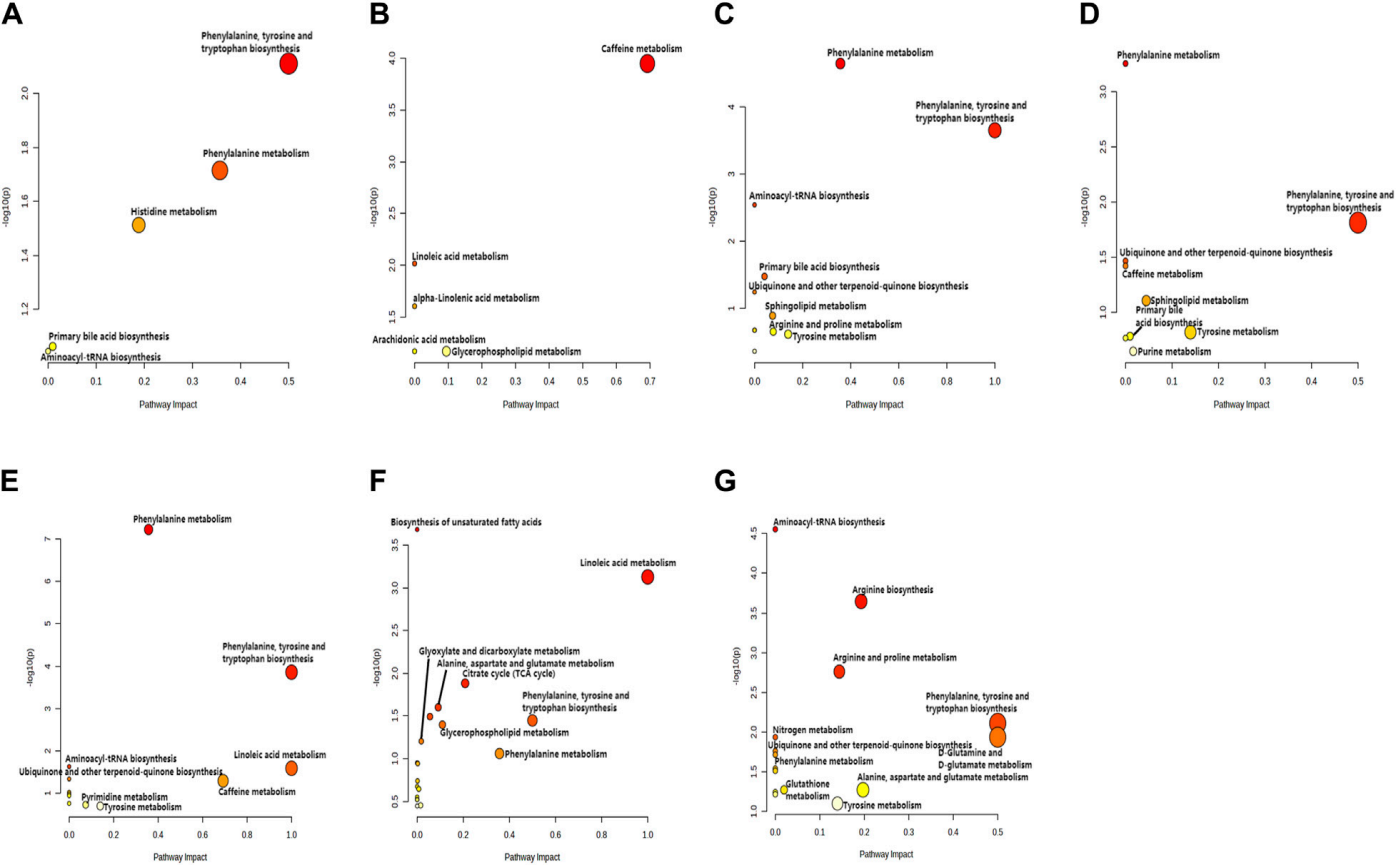
KEGG enrichment analysis of relevant pathways in different administration dose groups. Scatter plot of pathways enriched by important differential metabolites. The *X*-axis and size of a node indicate the influence of that path in the topological analysis pathway, and the *Y*-axis and color of a node indicate statistical significance. The doses were 0.5 mg panel **(A)**, 1 mg panel **(B)**, 2 mg panel **(C)**, 4 mg panel **(D)**, 7 mg panel **(E)**, 12 mg panel **(F)**, and 20 mg panel **(G)**.

**FIGURE 6:**
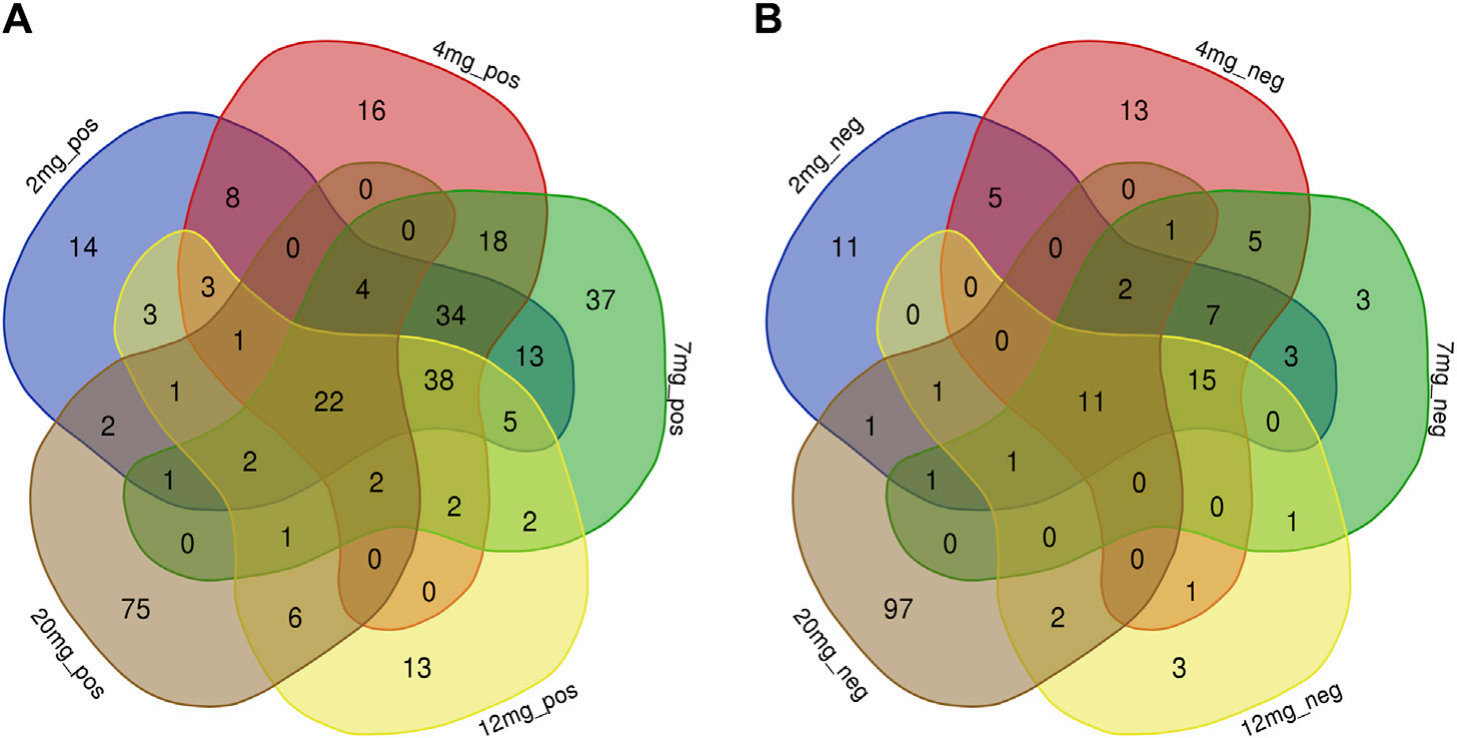
Common differential metabolites of five drug dose groups (2, 4, 7, 12, and 20 mg) in ESI+ and ESI-mode were screened by Venn diagram. Each colour area represents a dose group, numbers in individual colour areas indicate the number of different metabolites in that dose group, and numbers in overlapping colour areas indicate the number of metabolites that are common to two or more dose groups. The numbers in the centre of the graph indicate the number of metabolites shared among the five groups, and the Supplementary Table S1 lists the metabolites for each dose group. **(A)**: ESI + mode; **(B)** ESI-mode.

PLS-DA and OPLS-DA are supervised multivariate data analysis and belong to model methods. They use partial least squares regression to establish the relationship model between metabolite expression and sample type to reduce the data dimension. This supervised mode can better establish the relationship between samples and better obtain the different information between groups. In this study, PLS-DA and OPLS-DA results of each dose group showed that the pre-administration and the post-administration could be significantly separated in Electrospray ionization positive ion mode (ESI+) and Electrospray ionization negative ion mode (ESI-) (Figures 1A–G, 2A–G, 3A–G, 4A–G), suggesting that DS002 had A significant effect on the metabolism of the body. There was no over-fitting in the models of each group, and the prediction performance was good, indicating that the results were more accurate. Multivariate analysis was used to analyze the control and administration groups of different dose groups, and the differential metabolites between the control and administration groups were screened by setting the range of change fold and significance. We found that the levels of metabolites *in vivo* increased or decreased after administration of DS002. KEGG database was used to perform metabolic pathway enrichment analysis of differential metabolites in each dose group, and the pathways involved in each dose group were: 0.5 mg: Phenylalanine, tyrosine and tryptophan biosynthesis, Phenylalanine metabolism, Histidine metabolism, Primary bile acid biosynthesis, Aminoacyl-trna biosynthesis; 1 mg: Caffeine metabolism, Linoleic acid metabolism, α-linoleic acid metabolism, Arachidonic acid metabolism, Glycerophospholipid metabolism; 2 mg: Phenylalanine metabolism, Phenylalanine, tyrosine and tryptophan biosynthesis, Aminoacyl-tRNA biosynthesis, Primary bile acid biosynthesis, Ubiquinone and other terpene quinone biosynthesis, Sphingolipid metabolism, Arginine and proline metabolism, Tyrosine metabolism; 4 mg: Phenylalanine metabolism, Phenylalanine, tyrosine and tryptophan biosynthesis, Ubiquinone and other terpene quinone biosynthesis, Caffeine metabolism, Sphingolipid metabolism, Primary bile acid biosynthesis, Tyrosine metabolism, Purine metabolism; 7 mg: Phenylalanine metabolism, Phenylalanine, tyrosine and tryptophan biosynthesis, Linoleic acid metabolism, Caffeine metabolism, Aminoacyl-tRNA biosynthesis, Ubiquinone and other terpene quinone biosynthesis, Pyrimidine metabolism, Tyrosine metabolism; 12 mg: Unsaturated fatty acid biosynthesis, Linoleic acid metabolism, Phenylalanine, tyrosine and tryptophan biosynthesis, Phenylalanine metabolism, Citrate cycle (TCA), Alanine, aspartate and glutamate metabolism, Glyoxylate and dicarboxylic acid metabolism, Glycerophospholipid metabolism; 20 mg: Aminoacyl-tRNA biosynthesis, Phenylalanine, tyrosine and tryptophan biosynthesis, Arginine biosynthesis, Arginine and proline metabolism, Glutathione and glutamate metabolism, Glutathione metabolism, Ubiquinone and other terpene quinone biosynthesis, Phenylalanine metabolism, Alanine, aspartate and glutamate metabolism, Tyrosine metabolism (Figure 5). It is worth noting that aromatic amino acid metabolic pathways were found in the differential metabolic pathways of the six dose groups, and aromatic amino acids mainly included tyrosine, phenylalanine and tryptophan. Finally, the differential metabolites were summarized in the form of Venn diagram through the website Bioinformatics. Tryptophan was found in the differential metabolites of the five dose groups under ESI+ and ESI- modes (Figure 6). The compounds contained in each color region are listed in Supplementary Tables S1, S2. Therefore, aromatic amino acids were selected for the next targeted metabolomics analysis.

### 3.2 Targeted metabolomics for accurate quantification of aromatic amino acids and their metabolites

**FIGURE 7:**
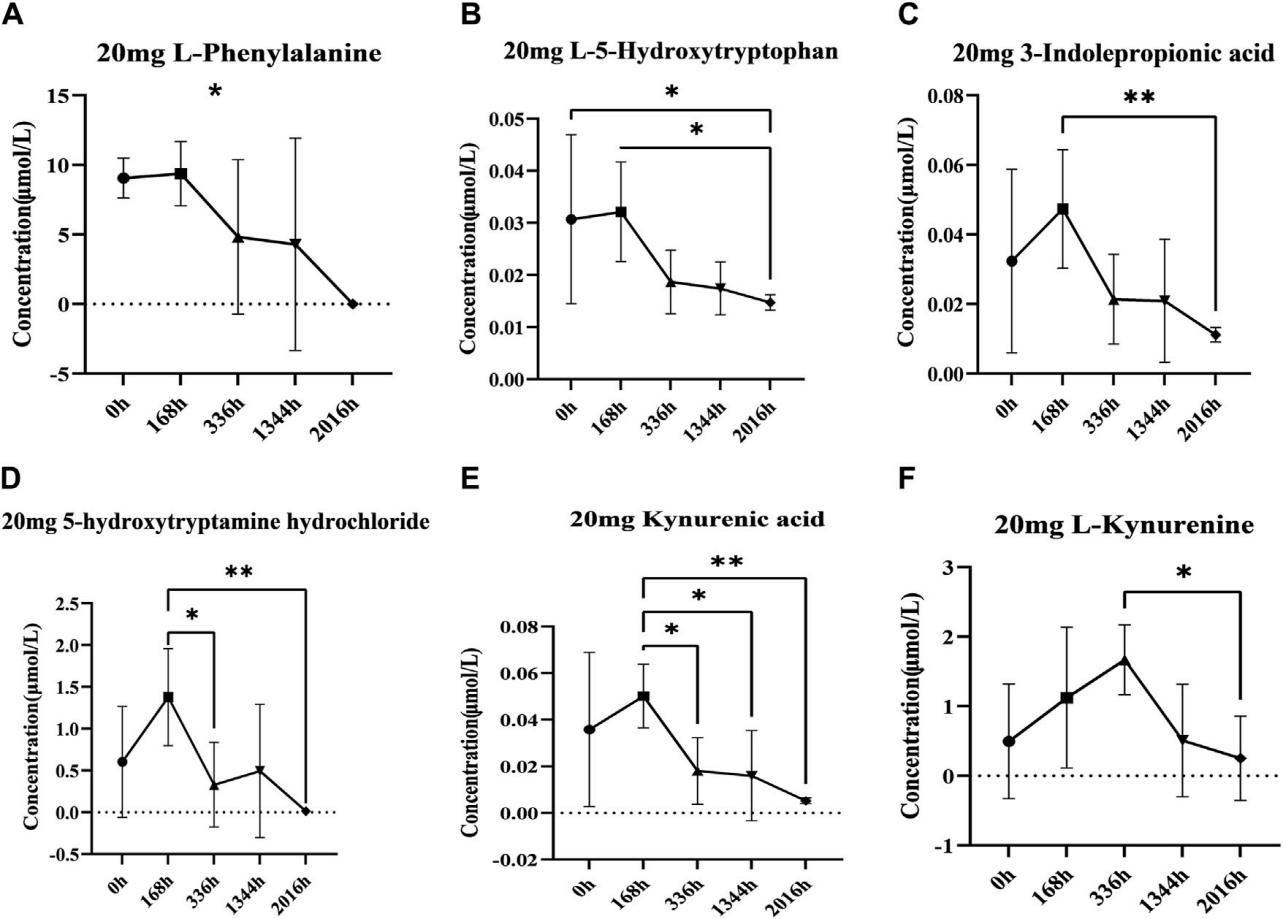
Analysis of concentration trends of important metabolites *in vivo*. Line graphs **(A)** L-phenylalanine, **(B)** L-5-hydroxytryptophan, **(C)** Indole-3-propionic acid, **(D)** 5-Hydroxytryptamine hydrochloride, **(E)** Kynurenic acid and **(F)** L-kynurenine describe the changing trend of each differential metabolite with administration time. * indicates *p* < 0.05.

Based on the results of non-targeted metabolomics experiments, we first established a targeted determination method for aromatic amino acids by liquid chromatography-tandem mass spectrometry. We used targeted metabolomics to accurately quantify aromatic amino acids and their metabolites. Statistical analysis of the targeted metabolomics data revealed that changes in important metabolites were statistically significant only in the highest administered dose group. The results showed that after administration of 20 mg, the concentrations of L-phenylalanine and 5-hydroxytryptophan decreased after administration; the concentrations of 3-indolepropionic acid, tryptamine hydrochloride, Kynurenic acid, and kynurenine increased first and then decreased (Figures 7A–F). The levels of these metabolites were then quantified in the placebo group and showed different trends between the placebo group and the treated group (Supplementary Figure S1). Therefore, we hypothesise that changes in these metabolites may be related to the effects of DS002 on pain, and that changes in these metabolites may stimulate the antisensory potential of the organism.

### 3.3 Changes in cartilage and bone metabolism-related indicators

**FIGURE 8:**
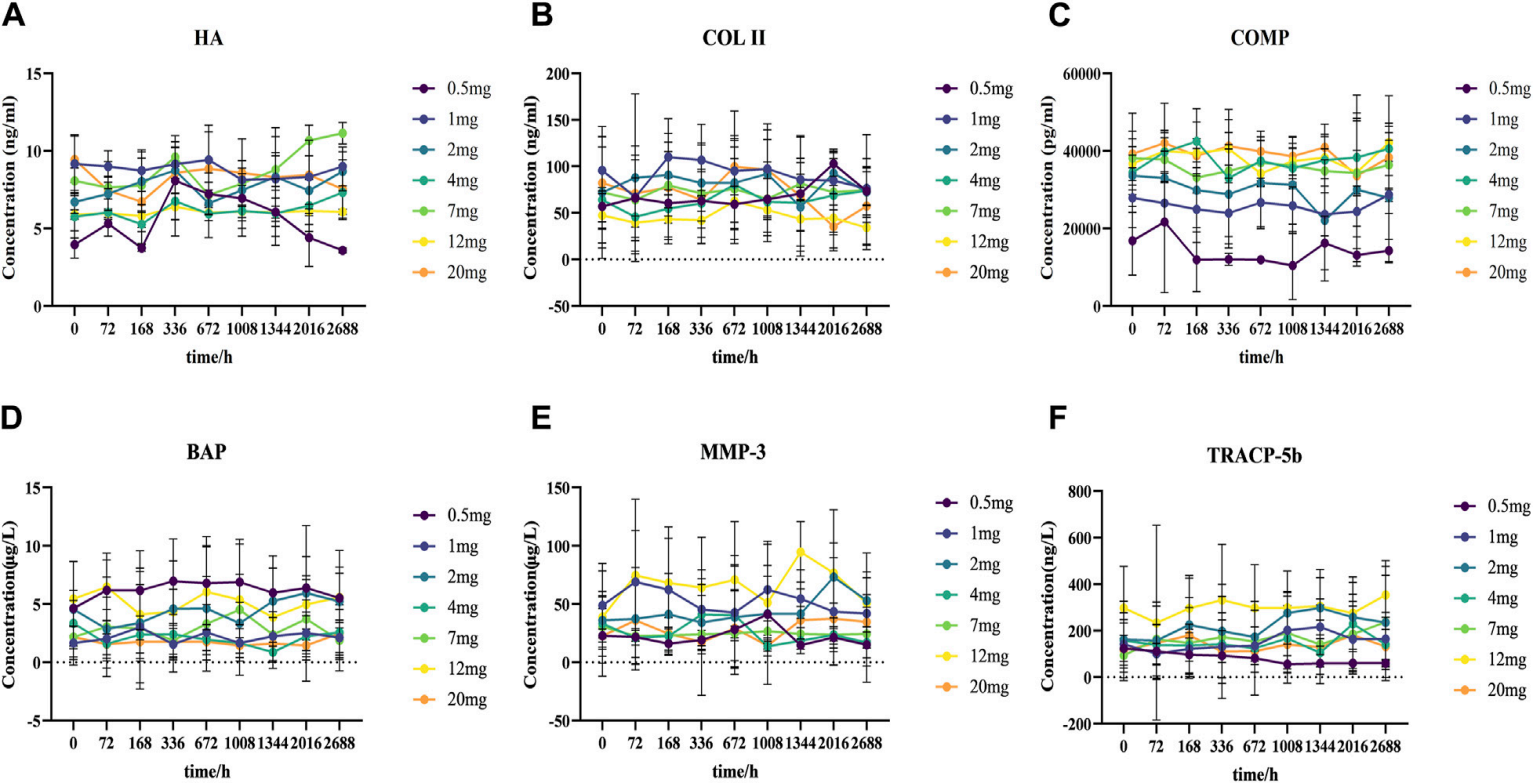
Analysis of the trend changes of markers related to cartilage and bone metabolism over time and dosing increments.Scatter plot **(A)** Hyaluronic acid (HA), **(B)** TypeⅡcollagen (COLII), **(C)** Cartilage oligomeric matrix protein (COMP), **(D)** Bone-specific alkaline phosphatase (BAP), **(E)** Matrix metalloproteinase 3 (MMP-3), and **(F)** tartrate-resistant acid phosphatase 5b (TRACP-5b) depict the trends of each marker as a function of administration time and dose.

Identifying the blood levels and distribution of different markers related to cartilage and bone metabolism can help to assess the risk of osteoarthritis. In our study, the concentrations of serum-related markers of cartilage and bone metabolism were determined before and after DS002 administration. The results showed that the serum concentrations of the six indicators related to cartilage and bone did not differ significantly with the changes in dose and time of administration (Figures 8A–F), indicating that DS002 had no significant effect on the markers related to cartilage and bone metabolism. So, we hypothesized that DS002 might not cause the serious adverse effects of joint necrosis and rapidly worsening osteoarthritis caused by the same target drug class.

## 4 Discussion

Pain is a common problem and is the main reason people seek medical care. But satisfactory clinical protocols for the treatment of pain remain elusive. Anti-NGF monoclonal antibody is a new class of analgesic drugs. In the studies that have been done on the treatment of pain with this class of drugs, it has shown high efficacy in the treatment of pain, far exceeding the efficacy of existing drugs, and is non-addictive. *In vitro* studies showed that DS002 successfully blocked the binding of NGF and TrkA receptors, thereby blocking the transmission of pain signals. DS002, as a novel analgesic drug targeting NGF, has the potential to become a new generation of drugs for treating chronic pain, nevertheless, previous studies have shown that co-targeted drugs of DS002 cause serious adverse effects on joints. In addition, the results of our Phase I clinical trial demonstrated that DS002 did not experience dose-limiting toxicity in terms of safety and did not meet dose-escalation termination criteria in the range of 0.5–20 mg administered (Ma et al., 2022). Five subjects developed arthralgia during the course of the trial, four subjects treated with DS002 (one in the 4.0 dose group, one in the 12.0 dose group, and two in the 20.0 mg dose group) and one in the placebo group, although the symptoms that developed were medically diagnosed to be mild, and all eventually resolved without intervention. No serious bone and joint events or peripheral neuropathy/sensory abnormalities were noted throughout the trial. Rapidly progressive arthritis (RPOA) was not observed even when the drug was administered at high doses. As this was the most prominent concern when the FDA shelved the development programme for similar anti-NGF drugs. Therefore, in the present study, we continued to investigate the effects of DS002 on cartilage and bone metabolism-related markers to better clarify whether it produces the same target drug-induced adverse effects in the joints and to discuss its safety in healthy subjects. Meanwhile, this study also explores the changes in metabolite levels in healthy subjects after taking DS002 from the perspective of blood metabolomics, and links the changes in metabolites to the mechanism of pain occurrence, to explore the reasons for the therapeutic effect of DS002, and to provide more scientific basis for advancing the marketing of DS002.

Previous Tanezumab clinical trial data showed joint adverse effects, including osteonecrosis and rapidly worsening osteoarthritis, in the same class as DS002 (Jayabalan and Schnitzer, 2017). Hyaluronic acid (HA) is the main component of the cartilage matrix, which can bind to CD44 receptors, thereby inhibiting the expression of interleukin (IL) 1β and reducing the production of matrix metalloproteinase 3 (MMP-3) (Karna et al., 2008). HA can enter the peripheral blood circulation through lymphatic circulation, and the expression level of HA is positively correlated with the severity of arthritis. Matrix metalloproteinases (MMPs) have been reported to play an essential role in the pathogenesis of osteoarthritis, as they are involved in the degradation of all matrices outside the cell except polysaccharides (Grillet et al., 2023; Murphy et al., 2002). Among them, MMP-3 is the most important protease involved in cartilage degradation, which can be regulated by IL-1 and TNF-α cytokines (Shi et al., 2022), followed by activation of other interstitial collagenases to degrade type II collagen (COL II), accelerates cartilage destruction. COL II is the main extracellular matrix protein in cartilage, and its degraded fragments will further accelerate the progression of osteoarthritis, promote the degradation of COL II, and accelerate the destruction of cartilage (Poole et al., 2002). Cartilage oligomeric matrix protein (COMP) is secreted by osteoblasts and synovium cells and is related to cartilage metabolism. After articular cartilage injury, cartilage matrix degradation produces COMP, which can be released into the blood (Maly et al., 2021). Bone alkaline phosphatase (BAP) is secreted by osteoblasts during the maturation stage and binds to bone matrix after being secreted to the extracellular space. It is a special sensitive index reflecting bone formation. In osteoporosis, bone calcification is insufficient. Osteoblasts proliferate actively and cannot transform into osteocytes. Osteoblasts proliferate actively and secrete a large amount of BAP into the blood. Tartrate resistant acid phosphatase 5b (TRACP-5b) is an enzyme secreted by activated bone calpain and is a good marker of bone resorption and osteoclast activity. Its concentration can be measured to understand bone metabolism status (Terpos et al., 2005). In this study, the concentrations of HA, MMP-3, COLII, COMP, BAP, TRACP-5b, which are related to cartilage and bone metabolism, did not change significantly over time in healthy subjects after treatment of different doses of DS002. These results indicated that the administration of monoclonal antibody DS002 did not cause fluctuations in the content of metabolites related to cartilage and bone metabolism, indicating that DS002 had no significant effect on the relevant parameters of cartilage and bone metabolism.

When pain occurs, a series of changes are reflected in the body’s metabolites. Specific blood, urine, and saliva biomarkers can be used to determine the degree of risk of chronic pain and can be used as prognostic markers of pain progression and treatment response (Hagedorn et al., 2021). In the present study, we measured the therapeutic effect of DS002 by investigating the blood metabolic profile. After DS002 administration, the body’s metabolism was significantly affected. The changes in metabolites involved in different metabolic pathways. According to previous studies, the metabolites in these metabolic pathways have been shown to be related to the occurrence and development of different pain. For example, researchers have found a link between amino acid metabolism and pain (Alexander et al., 2013; Teckchandani et al., 2021), the ratio of tryptophan to kynurenine is increased during back pain (Staats Pires et al., 2020), glutamate levels increase with the onset of fibromyalgia (Clos-Garcia et al., 2019), phosphocholine, alanine, and taurine are elevated in neuropathic pain (Finco et al., 2016). As the occurrence of pain is accompanied by a series of symptoms, different metabolic pathways can also indirectly affect pain. Studies have shown that the methionine metabolism pathway is related to sleep problems, and the glutathione metabolism pathway is related to obesity, and pain, sleep and obesity affect each other (Suzuki et al., 2015; Miettinen et al., 2019), so the imbalance of these two pathways will also affect pain. So we propose that the treatment of pain with DS002 may have a relationship to changes in these pathways. In our results, aromatic amino acid metabolic pathways account for the most significant proportion, such as the biosynthesis of phenylalanine, tyrosine, and tryptophan, as well as phenylalanine and tyrosine metabolism. These results may be relevant to the treatment of pain with DS002, because the amino acid metabolic pathway is related to the basal metabolic recovery of the body, and correct regulation reduces inflammatory response and affects nerve conduction. In addition, amino acids are the main substrate for producing neurotransmitters, and neurotransmitter-mediated pain regulation is the primary way.

Phenylalanine and tryptophan are essential amino acids for the human body and belong to the aromatic amino acids. Most phenylalanine in the body is oxidized to tyrosine, an amino acid associated with analgesic neurotransmitters, through the catalytic action of phenylalanine hydrogenase. The pathophysiological mechanisms underlying the onset and resolution of pain are intricate, and many different neurotransmitter systems are involved in the transmission, processing, and control of pain (Millan, 2002). In previous studies, Amino acid metabolites, including L-tryptophan, L-histidine, and L-tyrosine, were significantly decreased in the brain of pain model mice, suggesting that neuropathic pain may be promoted by the reduction of amino acids related to analgesic neurotransmitters. Therefore, we infer that the *in vivo* metabolic conversion of PHE to amino acids related to analgesic neurotransmitters would be involved in the therapeutic effect of DS002. Tryptophan is an essential amino acid for generating and maintaining proteins, muscles, enzymes, and neurotransmitters in the human body (Körtési et al., 2022). The tryptophan metabolism pathway includes the kynurenine, serotonin, and indole pathways. Changes in tryptophan levels can lead to imbalances in the synthesis and metabolism of downstream metabolites, which may be related to the pathological and physiological mechanisms of neurological and psychiatric diseases (Comai et al., 2020). Recent studies have shown that the dysregulation of the kynurenine pathway and the changes of metabolites in its pathway are associated with neurotoxicity and inflammation, and many metabolites are associated with chronic pain (Tanaka et al., 2021). Tryptophan enters the kynurenine metabolic pathway through indoleamine 2, 3-dioxygenase (IDO) and tryptophan 2, 3-dioxygenase (TDO) and generates kynurenine, which is irreversibly converted to kynurenic acid (KA) by kynurenine aminotransferase (KATs) (Schwarcz et al., 2012). KA is a neuroactive metabolite which belongs to the antiexcitotoxin and has neuroprotective effects (Fehér et al., 2019). The activity of KA at different targets is the primary mechanism by which it achieves specific functions. KA exerts its neuroprotective effect by antagonizing NMDA receptors (Birch et al., 1988), and previous studies have shown that marked reductions in neuroprotective metabolites occur in patients with psychiatric disorders (Athnaiel et al., 2022). KA interacts with g protein-coupled receptor 35 (GPR35) to indirectly reduce inflammation (Wirthgen et al., 2017), which is highly correlated with chronic pain. Thus, the neuroprotective and inflammatory response effects of KA suggest elevated levels of KA and other metabolites with similar properties when treating chronic pain symptoms.

In recent years, a great deal of research has been conducted on the role of 5-HT in chronic pain states.5-HT is metabolised in the body by: the small amount of tryptophan in the body is catalyzed by tryptophan hydroxylase (TPH) to generate 5-hydroxytryptophan (5-HTP). The aromatic L-amino acid decarboxylase interacts with the cofactor Pyridoxal 5-phosphate to convert 5-HTP to 5-hydroxytryptamine (5-HT) (Daubert and Condron, 2010). 5-HT, also known as serotonin, is an excitatory neurotransmitter in the human central nervous system, which causes the contraction of vascular smooth muscle, thereby potentially regulating pain (Taylor and Basbaum, 1995; Xue et al., 2023). Endogenous 5-HT was released in response to tissue injury in mast cells, platelets and endothelial cells, while multiple neuronal 5-HT receptors were detected in the periphery, suggesting that 5-HT can interfere with injurious transmission at peripheral sites (Bardin, 2011). Previous studies have also pointed to the complex relationship between 5-HT and pain, for example: intradermal administration of 5-HT to rats causes dose-dependent nociceptive hypersensitivity (Taiwo and Levine, 1992); plantar injections of 5-HT result in plantar oedema and intense flinching and licking behaviour (Sufka et al., 1992); In healthy volunteers, intradermal injections of low concentrations of 5-HT produced burning pain (Lischetzki et al., 2001), while injections into the mandibular occlusal muscle produced nociceptive hypersensitivity (Ernberg et al., 2000); Furthermore, In a carrageenan inflammatory pain model, administration of the 5-HT3 antagonist ICS-205930 by plantar administration at different times resulted in complete suppression of nociceptive hypersensitivity to foot inflammation, a process accompanied by 5-HT release (Eschalier et al., 1989). Taken together, these studies suggest that 5-HT release sensitises sensory nerve fibres in response to inflammation or tissue damage and promotes peripheral sensitisation through direct or indirect mechanisms. It has been shown that patients with chronic pain exhibit weaker endogenous pain inhibitory processes compared to pain-free individuals, and that the involvement of downward inhibition may represent an endogenous mechanism to prevent the chronicisation of pain (Ossipov et al., 2014). *In vivo*, the periaqueductal grey matter (PAG) is a brain region capable of activating endogenous pain inhibitory systems, and the PAG influences downstream pain modulation primarily through its interconnections with the rostral ventromedial medulla (RVM) (Ossipov et al., 2014), whereas the activation of downstream projections from the RVM induces the release of serotonin (Foo and Mason, 2003; Wei et al., 2010; Hossaini et al., 2012). 5-HT can exhibit both pro- and antinociception throughout, depending on the subtype of 5-HT receptor it activates, with activation of 5-HT1A, 5-HT1B, 5-HT1D, and 5-HT7 receptors tending to be antinociceptive, and 5-HT2A and 5-HT3 receptors tending to be proinjury sensory (Suzuki et al., 2004; Sasaki et al., 2006; Bannister et al., 2009; Dogrul et al., 2009); there is also a large body of research pointing to the inhibitory and facilitatory effects of the downstream 5-HT pathway on spinal neuronal excitability and pain-related behaviours, depending on the acute or persistent pain state (Bardin, 2011). In conclusion, these results suggest that 5-TH and the downstream 5-TH pathway play an important role in pain transmission in a complex pathological manner.

A minimal amount of tryptophan in the body can also be converted into indole and its derivatives, such as indole-3-acetic acid, indole-3-propionic acid, etc. under the action of the gut microbiota (Wei et al., 2021). Indole and its derivatives can contribute to the regulation of inflammatory responses (Venkatesh et al., 2014), which are highly correlated with the occurrence of pain. Therefore, indole and its derivatives can also affect the progression of pain. In addition, the gut microbiota is also an important regulator of visceral pain. In recent years, it has been found that the gut microbiota also plays a crucial role in chronic pain (Guo et al., 2019). In summary, these associations potentially suggest a correlation between changes in metabolite levels and the efficacy of DS002 in treating pain.

From the above, our findings suggest that DS002 has greater advantages in the treatment of pain compared with the existing drugs and the drugs in the same target class as DS002. Of course, the study has limitations. Our method of administration is a single dose in healthy people, which is different from clinical drug administration. Therefore, the application of DS002 in the clinical treatment of pain needs to be further explored.

## 5 Conclusion

Our results showed that DS002 caused the changes in the aromatic amino acid metabolic pathway in subjects treated with it. Among them, the changes in the concentration of L-phenylalanine, 5-hydroxytryptophan, 5-hydroxytryptamine, kynurenic acid, kynurenine, and indole-3-propionic acid may be related to the therapeutic effect of DS002 for treating pain. After administration, there were no significant changes in cartilage and bone metabolism indicators, and we preliminarily speculate that DS002 will not produce severe adverse reactions as the same target class of drugs. Our research provides a specific basis for DS002, and aromatic amino acids and their metabolites may serve as biomarkers for treating pain. Combined with the clinical results of the previous study of DS002 in our laboratory, it can more strongly demonstrate the enormous potential of anti-NGF drugs represented by DS002 in treating pain.

## Data Availability

The original contributions presented in the study are included in the article/Supplementary Material, further inquiries can be directed to the corresponding authors.
