# Supplementary material for: Effect of the novel anti-NGF monoclonal antibody DS002 on the metabolomics of pain mediators, cartilage and bone
Source: Front Pharmacol. 2024 Aug 12;15:1396790. doi: 10.3389/fphar.2024.1396790 (PMC11345146; doi:10.3389/fphar.2024.1396790)
Supplement: Supplementary file 1 [file DataSheet1.docx]

Supplementary Table 1: Compounds contained in each region of the positive ion model.

| Names | total | elements |
| --- | --- | --- |
| 12mg_pos.csv 20mg_pos.csv 2mg_pos.csv 4mg_pos.csv 7mg_pos.csv | 22 | Palmitoyl sphingomyelin、Glycerophosphocholine、Di-n-butyl phthalate、Linoleic acid、   1. Palmitoyl-2-linoleoyl-sn-glycero-3-phosphocholine、 2. 1-Heptadecanoyl-sn-glycero-3-phosphocholine、   Linoleoylcarnitine、  1-Docosahexaenoyl-2-stearoyl-sn-glycero-3-phosphocholine、  METHIONINE、Biliverdin、Piperine、HYPOXANTHINE、Phe-Phe、D-p-Chlorophenylalanine、   1. erythro-Sphingosine-1-phosphate、Phe-Trp、Erucamide、1-(1Z-Octadecenyl)-sn-glycero-3-phosphocholine 2. Decanoyl-L-Carnitine、ARGININE、5-OXO-L-PROLINE   Uric acid； CE20；LEHOTFFKMJEONL-UHFFFAOYSA-N |
| 12mg_pos.csv 2mg_pos.csv 4mg_pos.csv 7mg_pos.csv | 38 | MMV021013、  1-palmitoyl-2-hydroxy-sn-glycero-3-phosphoethanolamine、  Asp-Phe、Tyrosine、Glycocholic acid、PROLINE、"2,3-Dimethylhydroquinone"、  Tyramine;CE30;DZGWFCGJZKJUFP-UHFFFAOYSA-N、  Maltose、Hydrocortisone、Lauroyl-L-carnitine、Octanoylcarnitine、Linoleic acid methyl ester、L-Thyroxine、CREATINE、Dibutyl sebacate、  "1,2-DIDECANOYL-SN-GLYCERO-3-PHOSPHOCHOLINE"、Palmitoylcarnitine cation、Cortisone  1-Stearoyl-2-hydroxy-sn-glycero-3-phosphocholine、Palmitoleic acid ethyl ester、Glycoursodeoxycholic acid、Creatinine、Stachydrine、Beclomethasone、Palmitamide、Stearoyl-L-carnitine、"2,3',4,5'-Tetramethoxystilbene"、TRYPTOPHAN、Tri(butoxyethyl) phosphate、L-(+)-Lysine Monohydrate、1-Oleoyl-sn-glycero-3-phosphocholine、.gamma.-Dodecalactone、5-Methyl-5-phenylhydantoin、Glycohyodeoxycholic acid、Phthalic acid;CE20；XNGIFLGASWRNHJ-UHFFFAOYSA-N、Carnitine、5?-CHOLANIC ACID-3?_ 6?_7?-TRIOL |
| 20mg_pos.csv 2mg_pos.csv 4mg_pos.csv 7mg_pos.csv | 4 | L-Tryptophan、INOSINE、"cis-5,8,11,14-Eicosatetraenoic acid"、Oleoyl-L-carnitine |
| 12mg_pos.csv 20mg_pos.csv 2mg_pos.csv 4mg_pos.csv | 1 | Tris(1-chloro-2-propyl) phosphate |
| 12mg_pos.csv 20mg_pos.csv 2mg_pos.csv 7mg_pos.csv | 2 | Tributylamine、Oxprenolol |
| 12mg_pos.csv 20mg_pos.csv 4mg_pos.csv 7mg_pos.csv | 2 | Phenylacetylglutamine、Octanoyl-L-Carnitine |
| 2mg_pos.csv 4mg_pos.csv 7mg_pos.csv | 34 | 1-Lauroyl-2-hydroxy-sn-glycero-3-phosphocholine、Uric acid、3-Indoleacetic acid、Indole-3-carboxyaldehyde、  3-(2-Hydroxyethyl)indole、Tyr-W-MIF-1、Palmitelaidic acid、  Benzoic acid、  Hippuric acid;CE30;QIAFMBKCNZACKA-UHFFFAOYSA-N、Dopamine、Indole-3-carbinol、Lithocholenic Acid、Arachidoyl Ethanolamide、Risperidone、DL-Indole-3-lactic acid、DEOXYCARNITINE、Tetradecanoyl-L-Carnitine、Dimethyl sulfoxide、ISOLEUCINE、Chenodeoxycholic acid glycine conjugate、3-Indolepropionic acid、Acetyl-L-carnitine、KYNURENINE、Phenylacetic acid、Propionylcarnitine、Hexanoyl-L-carnitine、gamma-Glutamylleucine、His-Trp、L-Deprenyl、3-Methylpyrazole、Caffeine、  Phenylalanine;CE30;COLNVLDHVKWLRT-QMMMGPOBSA-N、Monoelaidin、L-Glutamine |
| 12mg_pos.csv 2mg_pos.csv 4mg_pos.csv | 3 | MMV020623、  1-pentadecanoyl-2-hydroxy-sn-glycero-3-phosphocholine、Betaine |
| 12mg_pos.csv 2mg_pos.csv 7mg_pos.csv | 5 | 7.alpha.-Hydroxy-3-oxo-4-cholestenoic acid、Tetradecylamine、"Dodeca-2(E),4(E)-dienoic acid"、  N-Octanoylsphingosine-1-phosphate、HISTIDINE |
| 20mg_pos.csv 2mg_pos.csv 7mg_pos.csv | 1 | "1-Hexadecyl-2-(5Z,8Z,11Z,14Z-eicosatetraenoyl)-sn-glycero-3-phosphocholine" |
| 12mg_pos.csv 20mg_pos.csv 2mg_pos.csv | 1 | LEUCINE |
| 12mg_pos.csv 4mg_pos.csv 7mg_pos.csv | 2 | MMV668727、Diethyl-phthalate |
| 12mg_pos.csv 20mg_pos.csv 7mg_pos.csv | 1 | GLUTAMIC ACID |
| 2mg_pos.csv 4mg_pos.csv | 8 | DL-Pipecolinic acid、Taurocholic acid、Taurocholate、  Glutamate; CE20; WHUUTDBJXJRKMK-VKHMYHEASA-N、  (25S)-7-Dafachronic acid、  "2-Piperidinecarboxylic acid, 1-(3,3-dimethyl-1,2-dioxopentyl)-, (1R)-1-(3-aminophenyl)-3-(3,4-dimethoxyphenyl)propyl ester, (2S)-"、  Dimethyl sulfoxide  Nootkatone |
| 2mg_pos.csv 7mg_pos.csv | 13 | 2-Indolinone、Citrulline、URIDINE、  (S)-N-(1-Amino-3-methyl-1-oxobutan-2-yl)-1-(5-fluoropentyl)-3-(4-fluorophenyl)-1H-pyrazole-5-carboxamide、  Xanthine; CE20; LRFVTYWOQMYALW-UHFFFAOYSA-N  N-Desmethyltrimipramine、"1,7-Dimethylxanthine"、Indole、Pantothenic acid、D-erythro-N-stearoylsphingosine、Phe-Glu、MMV658993、Ricinoleic acid methyl ester |
| 12mg_pos.csv 2mg_pos.csv | 3 | "N,N-Dimethyldodecylamine N-oxide"、Acetyl-L-Carnitine、".alpha.,.beta.-Thujone" |
| 20mg_pos.csv 2mg_pos.csv | 2 | Phenylacetylglutamine;CE20;JFLIEFSWGNOPJJ-JTQLQIEISA-N、1-Stearoyl-sn-glycero-3-phosphocholine |
| 4mg_pos.csv 7mg_pos.csv | 18 | d-ribo Phytosphingosine 1-Phosphate、Theobromine、4-Hydroxyquinoline、D-ORNITHINE、Isobutyryl-L-carnitine、Ethyl pentadecanoate、Docosahexaenoic acid methyl ester、cis-Vaccenic acid、Oleamide、"rac-N,O-Didesmethylvenlafaxine"、(S)-Perillic acid、gamma-Glutamyltyrosine、4-Hydroxy-6-methyl-2-pyrone、Dihomo-.gamma.-linolenic acid methyl ester、Lithospermic acid、N-Oleoylglycine、"2,3,5,6-Tetramethylpyrazine"、Pyridine |
| 12mg_pos.csv 7mg_pos.csv | 2 | Promethazine N-oxide、Tetraglyme |
| 12mg_pos.csv 20mg_pos.csv | 6 | Dimethyl suberate、Bis(2-ethylhexyl) adipate、Phylloporphyrin、Digoxigenin monodigitoxoside、5?-CHOLANIC ACID-3?-OL-6_7-DIONE、  PHENYLALANINE |
| 2mg_pos.csv | 14 | 17.beta.-Hydroxy-17.alpha.-methyl-5.alpha.-androstan-1-en-3-one、Ornithine、N-Desmethyltramadol、Dyphylline、Testosterone、Monoisobutyl phthalate、Pracinostat、Drospirenone、Toddalolactone、Rabenzazole、(R)-Butyrylcarnitine、(-)-Riboflavin、MMV028694、Corticosterone |
| 4mg_pos.csv | 16 | 3-(1-Pyrazolyl)-alanine、Praziquantel、Docosatrienoic acid、21-Deoxycortisol、"21-Hydroxy-5.beta.-pregnane-3,11,20-trione"、D-sphingosine、Glu-Phe、L-Histidine、"Androstane-3,17-diol"、8-Hydroxyadenosine、Isorhoifolin、Dexpanthenol、Theophylline; CE20; ZFXYFBGIUFBOJW-UHFFFAOYSA-N、  Pyridoxine; CE40; LXNHXLLTXMVWPM-UHFFFAOYSA-N  2-Aminoacetophenone、D-Pantothenic acid |
| 7mg_pos.csv | 37 | Glutamic acid、Lauric acid diethanolamide、Phe-Leu、Tris(2-chloropropyl) phosphate、5-Amino-2-methoxyphenol、"3,4-Dimethylbenzaldehyde"  Uric acid; CE10; LEHOTFFKMJEONL-UHFFFAOYSA-N、Gambogic acid、  GUANOSINE、Justicidin B、cis-12-Octadecenoic acid methyl ester、Telmisartan、Dehydroepiandrosterone (DHEA)、4-Methylcyclohexylamine、Pro-Phe、URACIL、Hydroxy-cholenoic acid、LysoPE(20:4)、"N2,N2-Dimethylguanosine"、beta-Hydroxyisovaleric acid、Gly-Arg-Gly-Asp-Ser-Pro、Hematoporphyrin_I、N-(4-Cyanophenyl)glycine、  ?-linolenic acid、Thiazolidine-4-carboxylic acid、D-erythro-Sphingosine C-20、  Hypoxanthine; CE20; FDGQSTZJBFJUBT-UHFFFAOYSA-N、DDAO、VALINE、PIPECOLIC ACID、Hexaethylene glycol、Hydroxybutorphanol、PIPECOLINIC ACID、"3,5-Di-tert-butyl-4-hydroxybenzoic acid"、3-Ketocholanic Acid、Chorismic acid、CHOLESTERYL ACETATE |
| 12mg_pos.csv | 13 | Di(2-nonyl) phthalate、Nifenazone、Oleoyl-L-Carnitine、2-Phenylacetamide、Venlafaxine、Alprenolol、N-Methyl-2-pyrrolidone、CYSTINE、Dioctyl Phthalate、Isophorone、Octocrylene、Histamine-trifluoromethyltoluide、Sclareolide |
| 20mg_pos.csv | 75 | Phytosphingosine 1-phosphate、"C13:0,DC FA(1) (Tridecanedicarboxylic acid)"、PALMITOLEIC ACID、Propiolate、5-Methylcytidine、"C16:1,OH FA(2)"、D(-)-Fructose、Glucose、"5.alpha.-Pregnan-3.alpha.,17-diol-20-one 3-sulfate"、Catechol sulfate、Taurine、"C18:2,DC FA (Octadecadienedicarboxylic acid)"、"9Z, 11E-Linoleic acid"、N-Acetyl-D-Valine、Vitamin K1、LAUROYLCARNITINE、3-Indoleacrylic acid、L(+)-Arginine hydrochloride、Di(2-ethylhexyl)phthalate (DEHP)、3-O-Methyluridine、5.alpha.-Androstan-3.beta.-ol-17-one sulfate、C12:2,OH FA"、Levulinic acid、C10:0 AC、C24:4 FA、C22:1 FA (Catelaidic acid)、LysoPI(18:1)、Tryptophan、Ethyl myristate、PC(16:0/0:0)、Glycodeoxycholic acid、C12:1 FA(1) (Dodecenoic acid)、C11:1 FA、Hydroxyphenethylamine、Oleoyl Ethyl Amide、"C18:0,DC FA(2)"、Galactinol、Arachidonic acid、  Phthalic acid; CE30; XNGIFLGASWRNHJ-UHFFFAOYSA-N、Oxalacetic acid、16a-hydroxy DHEA 3-sulfate、FERULATE、  Tetrahydroaldosterone-3-glucuronide(2)、3-Aminopentanoic acid、Ketodeoxycholic acid(3)、L-Proline、MG(15:0)(1)、C20:0 FA、Pyridoxamine-5'-phosphate、RAC-GLYCEROL 1-MYRISTATE、O-Benzyl-L-serine、Phthalic acid、  Stearic acid、"1,2-dioleoyl-sn-glycero-3-phosphatidylcholine"、"2,4-Di-tert-butylphenol"、C24:4 FA (Tetracosatetraenoic acid)、1-Palmitoyl-2-hydroxy-sn-glycero-3-phosphoethanolamine、3-tert-Butyl-4-hydroxyanisole、Isoleucine、Acetyl-DL-carnitine、C20:1 FA (Eicosenoic acid)、5.alpha.-Androstan-17.beta.-ol-3-one sulfate、MG(18:0)、Cholesterol glucuronide、4-Androsten-17.beta.-ol-3-one sulfate、L-Alanylglycine、"4,6-Diamino-5-(formylamino)pyrimidine (FAPy-Adenine)"、  "1,2-Dihexadecanoyl-sn-glycero-3-phosphocholine"、C22:4 FA、Sulfolithocholic acid、TYROSINE、4-Guanidinobutyric acid、"1,2-Dilinoleoyl-sn-glycero-3-phosphocholine"、L-Phenylalanine |

Supplementary Table 2: Compounds contained in each region in the negative ion mode.

| Names | total | elements |
| --- | --- | --- |
| 12mg_neg.csv 20mg_neg.csv 2mg_neg.csv 4mg_neg.csv 7mg_neg.csv | 11 | Phenylacetylglutamine、Oxalacetic acid  "5.alpha.-Pregnan-3.alpha.,17-diol-20-one 3-sulfate"、  Phthalic acid、Stearic acid、"2,4-Di-tert-butylphenol"、  1-Palmitoyl-2-hydroxy-sn-glycero-3-phosphoethanolamine、L-Tryptophan、5.alpha.-Androstan-17.beta.-ol-3-one sulfate、D-erythro-Sphingosine-1-phosphate、L-Phenylalanine |
| 12mg_neg.csv 2mg_neg.csv 4mg_neg.csv 7mg_neg.csv | 15 | Glycoursodeoxycholic acid、Chenodeoxycholic acid glycine conjugate、Uric acid、Palmitic acid、Tetrahydrocortisol glucuronide(2)、1-Oleoyl-sn-glycero-3-phosphoethanolamine、Citric acid、LINOLEATE、Androsterone sulfate(2)、Indoxyl sulfate、CMPF、Inosine、Epitestosterone sulfate、Cholic acid、Androsterone sulfate |
| 20mg_neg.csv 2mg_neg.csv 4mg_neg.csv 7mg_neg.csv | 2 | 16a-hydroxy DHEA 3-sulfate、4-Androsten-17.beta.-ol-3-one sulfate |
| 12mg_neg.csv 20mg_neg.csv 2mg_neg.csv 7mg_neg.csv | 1 | Isoleucine |
| 2mg_neg.csv 4mg_neg.csv 7mg_neg.csv | 7 | 16-Phenoxytetranorprostaglandin E2、Glycocholic acid、Phe-Ile、Tauroursodeoxycholic acid、Deoxycholic acid 3-glucuronide(3)、3-Hydroxyphenylalanine、Trans-Vaccenic acid |
| 20mg_neg.csv 2mg_neg.csv 7mg_neg.csv | 1 | Arachidonic acid |
| 12mg_neg.csv 20mg_neg.csv 2mg_neg.csv | 1 | D(-)-Fructose |
| 20mg_neg.csv 4mg_neg.csv 7mg_neg.csv | 1 | Ethyl myristate |
| 2mg_neg.csv 4mg_neg.csv | 5 | Deoxycholic acid 3-glucuronide(1)、  D-erythro-Sphinganine-1-phosphate、.gamma.-Hydroxybutyric acid、2-Hydroxy-4-methylpentanoic acid、Taurocholic acid(2) |
| 2mg_neg.csv 7mg_neg.csv | 3 | Pregnanolone sulfate、Vaccenic acid、LysoPE(20:4) |
| 20mg_neg.csv 2mg_neg.csv | 1 | Phe-Phe |
| 4mg_neg.csv 7mg_neg.csv | 5 | DL-Lactic acid、Asp-Phe、Chenodeoxycholic Acid(1)、DL-Pyroglutamic acid、3-(4-Hydroxyphenyl)lactic acid |
| 12mg_neg.csv 4mg_neg.csv | 1 | (3.beta.)-Allopregnanolone sulfate |
| 12mg_neg.csv 7mg_neg.csv | 1 | 4-Chloro-L-phenylalanine |
| 12mg_neg.csv 20mg_neg.csv | 2 | O-Benzyl-L-serine、3-tert-Butyl-4-hydroxyanisole |
| 2mg_neg.csv | 11 | 1-Oleoyl-L-.alpha.-lysophosphatidic acid、  "9,10-Dihydroxy-12Z-octadecenoic acid"、Pinolenic acid、Mannose、1H-Indole-3-propanoic acid、Salicylic Acid、delta4-Dafachronic acid、Butylparaben、Indolelactic acid、"1,3-Cyclohexanedicarboxylic acid"、  "4-Pregnen-17.alpha., 20.beta.-diol-3-one-20-sulfate" |
| 4mg_neg.csv | 13 | lauryl sulfate、Dehydroisoandrosterone sulfate、Palmitoleic acid、Norleucine、Indole-3-propionic acid、3-Hydroxyvaleric acid、Baicalin、Pseudouridine、  "12,13-Dihydroxy-9Z-octadecenoic acid"、a-Linolenic acid、Murocholic acid、Taurocholic acid、  "N,N,Diacetyl-Lys-DAla-DAla" |
| 7mg_neg.csv | 3 | y-Linolenic acid、3-Hydroxybutyric acid、Oxypurinol |
| 12mg_neg.csv | 3 | Tyrosine、5-Phenylvaleric acid、Oleic acid |
| 20mg_neg.csv | 97 | Phytosphingosine 1-phosphate、"cis-5,8,11,14-Eicosatetraenoic acid"、"C13:0,DC FA(1) (Tridecanedicarboxylic acid)"、GLUTAMIC ACID、Phthalic acid;、CE30、 XNGIFLGASWRNHJ-UHFFFAOYSA-N、Palmitoyl sphingomyelin、PALMITOLEIC ACID、FERULATE、Propiolate、Tetrahydroaldosterone-3-glucuronide(2)、Glycerophosphocholine、3-Aminopentanoic acid、5-Methylcytidine、Di-n-butyl phthalate、Ketodeoxycholic acid(3)、"C16:1,OH FA(2)"、Glucose、HYPOXANTHINE  、L-Proline、MG(15:0)(1)、C20:0 FA、LEUCINE、Catechol sulfate、Taurine  、"C18:2,DC FA (Octadecadienedicarboxylic acid)"、"9Z, 11E-Linoleic acid"、Pyridoxamine-5'-phosphate、Linoleic acid、RAC-GLYCEROL 1-MYRISTATE、N-Acetyl-D-Valine、Vitamin K1、LAUROYLCARNITINE  、3-Indoleacrylic acid、Phylloporphyrin、Tributylamine、"1-Hexadecyl-2-(5Z,8Z,11Z,14Z-eicosatetraenoyl)-sn-glycero-3-phosphocholine"、D-p-Chlorophenylalanine、L(+)-Arginine hydrochloride、"1,2-dioleoyl-sn-glycero-3-phosphatidylcholine"、C24:4 FA (Tetracosatetraenoic acid)、Digoxigenin monodigitoxoside、1-Palmitoyl-2-linoleoyl-sn-glycero-3-phosphocholineDi(2-ethylhexyl)phthalate (DEHP)、3-O-Methyluridine、Acetyl-DL-carnitine、C20:1 FA (Eicosenoic acid)、1-Stearoyl-sn-glycero-3-phosphocholine、5.alpha.-Androstan-3.beta.-ol-17-one sulfate、"C12:2,OH FA"、1-Heptadecanoyl-sn-glycero-3-phosphocholine、Levulinic acid、MG(18:0)、Dimethyl suberate、Cholesterol glucuronide、Phe-Trp、C10:0 AC、5?-CHOLANIC ACID-3?-OL-6_7-DIONE、C24:4 FA、C22:1 FA (Catelaidic acid)、Linoleoylcarnitine、INOSINE、Octanoyl-L-Carnitine、LysoPI(18:1)、L-Alanylglycine、Tryptophan、"4,6-Diamino-5-(formylamino)pyrimidine (FAPy-Adenine)"、PC(16:0/0:0)、Erucamide、Glycodeoxycholic acid、"1,2-Dihexadecanoyl-sn-glycero-3-phosphocholine"、  1-(1Z-Octadecenyl)-sn-glycero-3-phosphocholine、C12:1 FA(1) (Dodecenoic acid)、Oleoyl-L-carnitine、Decanoyl-L-Carnitine、C11:1 FA、C22:4FA、Phenylacetylglutamine;CE20;JFLIEFSWGNOPJJ-JTQLQIEISA-N、1-Docosahexaenoyl-2-stearoyl-sn-glycero-3-phosphocholine、METHIONINE、ARGININE、Sulfolithocholic acid、Tris(1-chloro-2-propyl) phosphate、Biliverdin、Bis(2-ethylhexyl) adipate、Hydroxyphenethylamine、PHENYLALANINE、TYROSINE、4-Guanidinobutyric acid、Oxprenolol、Oleoyl Ethyl Amide、"1,2-Dilinoleoyl-sn-glycero-3-phosphocholine"、5-OXO-L-PROLINE、"C18:0,DC FA(2)"、Piperine、Uric acid; CE20; LEHOTFFKMJEONL-UHFFFAOYSA-N、Galactinol |


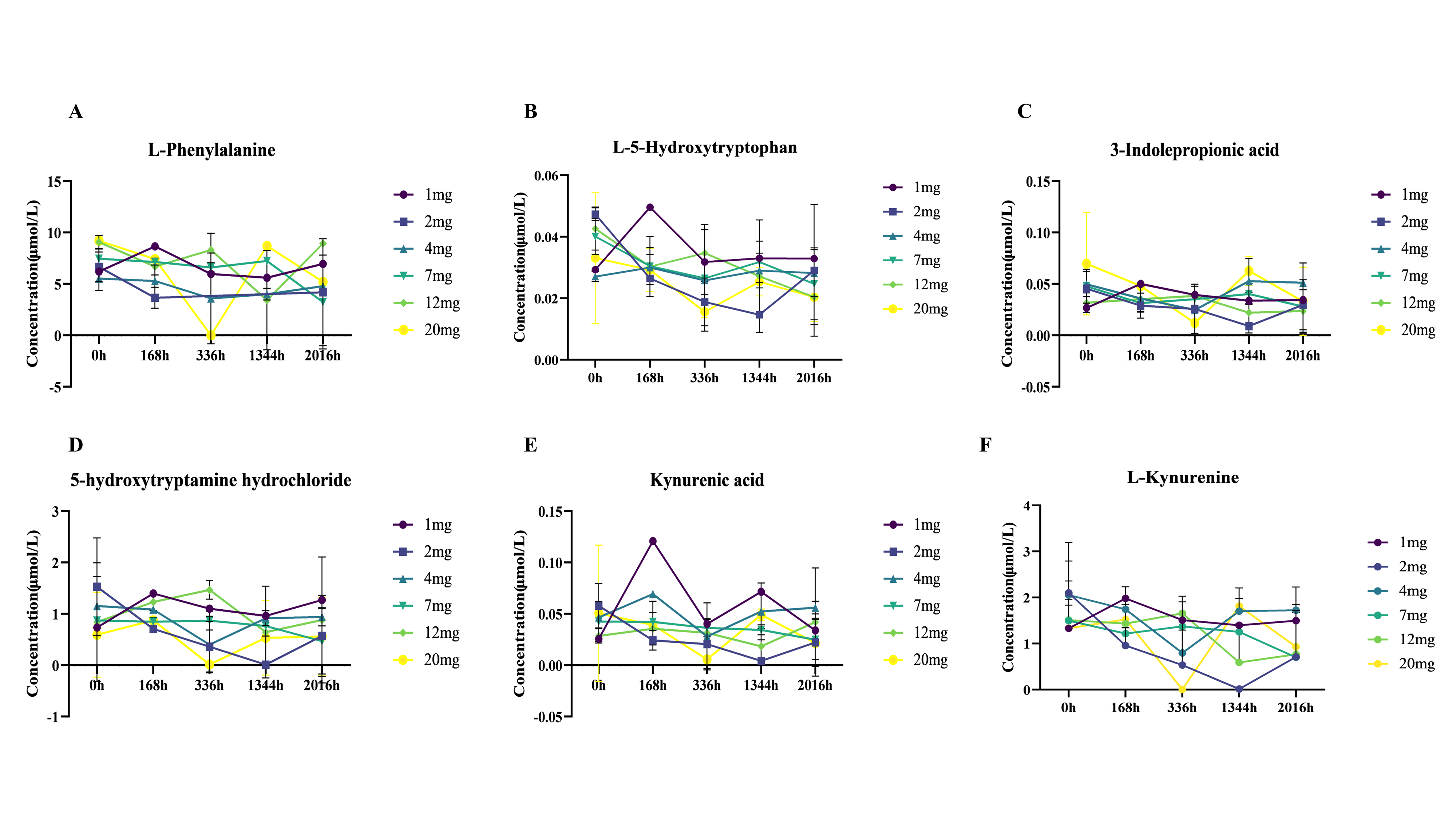


Supplementary Fig.1: Trend analysis of important metabolites in the placebo group at different administered doses.A. L-phenylalanine, B. L-5-hydroxytryptophan, C. indole-3-propionic acid, D. 5-hydroxytryptophan hydrochloride, E. kynurenine, F. L-kynurenine.
